# Trajectory of vitamin D status during pregnancy in relation to neonatal birth size and fetal survival: a prospective cohort study

**DOI:** 10.1186/s12884-018-1683-7

**Published:** 2018-02-13

**Authors:** Linnea Bärebring, Maria Bullarbo, Anna Glantz, Lena Hulthén, Joy Ellis, Åse Jagner, Inez Schoenmakers, Anna Winkvist, Hanna Augustin

**Affiliations:** 1 The Department of Internal Medicine and Clinical Nutrition, Sahlgrenska Academy, University of Gothenburg, Box 459, 405 30 Gothenburg, Sweden; 2 Södra Älvsborg Hospital, Borås, Sweden; 3 The Department of Obstetrics and Gynecology, Sahlgrenska Academy, University of Gothenburg, Gothenburg, Sweden; 4 Department of Antenatal Care, Närhälsan, Primary Care, Gothenburg, Sweden; 5 Department of Antenatal Care, Närhälsan, Primary Care, Södra, Bohuslän, Sweden; 6 MRC Human Nutrition Research, Nutrition and Bone Health Group, Cambridge, UK; 7 The Department of Medicine, Faculty of Medicine and Health Sciences, University of East Anglia, Norwich, UK

**Keywords:** Vitamin D, 25-hydroxyvitamin D, Small for gestational age, Low birth weight, Preterm delivery, Miscarriage, Intrauterine fetal death

## Abstract

**Background:**

We investigated the associations between vitamin D status in early and late pregnancy with neonatal small for gestational age (SGA), low birth weight (LBW) and preterm delivery. Furthermore, associations between vitamin D status and pregnancy loss were studied.

**Methods:**

Serum 25-hydroxyvitamin D (25OHD) was sampled in gestational week ≤ 16 (trimester 1 (T1), *N* = 2046) and > 31 (trimester 3 (T3), *N* = 1816) and analysed using liquid chromatography tandem mass spectrometry. Pregnant women were recruited at antenatal clinics in south-west Sweden at latitude 57–58°N. Gestational and neonatal data were retrieved from medical records. Multiple gestations and terminated pregnancies were excluded from the analyses. SGA was defined as weight and/or length at birth < 2 SD of the population mean and LBW as < 2500 g. Preterm delivery was defined as delivery < 37 + 0 gestational weeks and pregnancy loss as spontaneous abortion or intrauterine fetal death. Associations between neonatal outcomes and 25OHD at T1, T3 and change in 25OHD (T3-T1) were studied using logistic regression.

**Results:**

T1 25OHD was negatively associated with pregnancy loss and 1 nmol/L increase in 25OHD was associated with 1% lower odds of pregnancy loss (OR 0.99, *p* = 0.046). T3 25OHD ≥ 100 nmol/L (equal to 40 ng/ml) was associated with lower odds of SGA (OR 0.3, *p* = 0.031) and LBW (OR 0.2, *p* = 0.046), compared to vitamin D deficiency (25OHD < 30 nmol/L, or 12 ng/ml). Women with a ≥ 30 nmol/L increment in 25OHD from T1 to T3 had the lowest odds of SGA, LBW and preterm delivery.

**Conclusions:**

Vitamin D deficiency in late pregnancy was associated with higher odds of SGA and LBW. Lower 25OHD in early pregnancy was only associated with pregnancy loss. Vitamin D status trajectory from early to late pregnancy was inversely associated with SGA, LBW and preterm delivery with the lowest odds among women with the highest increment in 25OHD. Thus, both higher vitamin D status in late pregnancy and gestational vitamin D status trajectory can be suspected to play a role in healthy pregnancy.

## Background

Placental pathology is often found in pregnancies complicated by intrauterine growth restriction, preeclampsia or intrauterine fetal death. It is associated with inadequate invasion of extravillous trophoblasts and inadequate angiogenesis, with insufficient conversion of arterial spiral arteries in the decidua [1]. These processes are complex and normal development is dependent on several factors. Vitamin D status (measured as 25-hydroxyvitamin D (25OHD)) of pregnant women has been inversely associated with adverse gestational outcomes and associated with uteroplacental dysfunction [2].

We have previously shown that higher vitamin D status in late pregnancy and larger increase in vitamin D status during pregnancy is associated with lower risk of preeclampsia [3]. Associations have also been shown between vitamin D insufficiency (< 50 nmol/L) and increased risk of infant small for gestational age (SGA) and low birth weight (LBW) [4]. Circulating concentrations of 25OHD below 25 or 30 nmol/L have been associated with 50–300% increased odds of SGA, compared to higher concentrations [5–7]. Also, maternal 25OHD < 28 nmol/L in late, but not early, pregnancy have been associated with lower infant birth size and shorter gestational length in a smaller longitudinal study [8]. However, there is also limited evidence for a U-shaped association where high 25OHD concentrations (> 80 nmol/L) may be related to higher risk of SGA [9]. Thus, the association between vitamin D status and neonatal birth size is not clear and warrants further investigation. A recent meta-analysis of 10 studies concluded that 25OHD concentrations < 50 nmol/L were associated with an approximately 30% increased risk of preterm delivery [10]. Bodnar et al. found that early pregnancy 25OHD concentrations < 75 nmol/L were associated with higher risk of both medically indicated and spontaneous preterm delivery [11]. Lower vitamin D status has also been associated with a medical history of recurrent miscarriage [12] but only two prospective studies have investigated this, without finding that lower 25OHD concentrations increases the risk of pregnancy loss [5, 13]. Both these prospective studies had relatively few cases of pregnancy loss and may therefore have been insufficiently powered to study this association. Thus, the association between vitamin D status and risk of miscarriage needs further investigation.

To our knowledge, no previous study has related 25OHD concentration in both early and late pregnancy to neonatal outcomes related to placental dysfunction. Therefore, it has not been possible to ascertain whether the associations at implantation in early pregnancy differ from those during the fetal growth spurt in late pregnancy.

Our objectives were to study the associations between vitamin D status in both early and late pregnancy, as well as change in vitamin D status during pregnancy with neonatal SGA, LBW and preterm delivery. Associations between vitamin D status in early pregnancy and pregnancy loss were also studied.

## Methods

The GraviD study is a prospective cohort study, conducted in parts of the Västra Götaland region in the southwest of Sweden, at latitude 57–58°N [3]. Pregnant women were recruited from gestational week 4 during fall 2013 and spring 2014, when registering at one of the participating antenatal care units. The only exclusion criterion was pregnancy exceeding 16 gestational weeks at inclusion. Gestational age at delivery and data collection was determined by routine ultrasound in the second trimester, but gestational age at inclusion was based on the date of the last menstrual period. Women who terminated the pregnancy (*N* = 31), were lost to follow-up (*N* = 13) or carried more than one foetus (*N* = 26) were excluded from the present analyses. Pregnancy termination was mostly due to fetal malformations. Women who were lost to follow-up had moved and their medical records could not be retrieved. This study was conducted according to the Declaration of Helsinki and all procedures were approved by the Regional Ethics Committee in Gothenburg, Sweden. Written informed consent was obtained from all participants.

### Outcomes

SGA was defined as either weight or length at birth below 2 SD of the gender-specific population mean [14]. LBW was defined as weight at birth < 2500 g. Preterm delivery was defined as delivery before gestational week+days 37 + 0, including both induced and spontaneous preterm delivery. Pregnancy loss was defined as both spontaneous abortions from gestational week 4 and intrauterine fetal death (IUFD). Late pregnancy loss was defined as spontaneous abortion at gestational week ≥ 14 + 0, including IUFD. IUFD was defined as pregnancy loss at gestational week ≥ 22 + 0. Pregnancy loss before gestational week 22 was based on self-report data and medical records to verify miscarriage were not available. Information on IUFD was collected from medical records from the obstetrics care.

### Exposure

Maternal blood samples were collected from each participant at two time points; before gestational week 16 (predominantly week 8–12, first trimester, T1) and after gestational week 31 (predominantly week 32–35, third trimester, T3). Non-fasting venous blood samples were drawn in gel serum separating tubes and centrifuged for 10 min within 2 h of sampling. Serum was stored at − 70 °C until analysis of 25OHD. Analyses were performed by liquid chromatography tandem mass spectrometry (LC-MS/MS, Mass spectrometer API 4000) by the clinical chemistry laboratory in Region Skåne, Sweden, certified by the Vitamin D External Quality Assessment Scheme [15]. The LC-MS/MS method has a measuring range of 6–450 nmol/L for 25OHD_3_ and of 6–225 nmol/L for 25OHD_2_. The inter-assay coefficient of variation is 6% at 40 nmol/L for both 25OHD_3_ and 25OHD_2_. Sampling and laboratory analyses have been described previously [3]. Maternal serum T1 and T3 samples were analysed in sequence. At T1 and T3, participants answered questionnaires regarding lifestyle factors and background data. Neonatal and gestational data were obtained from antenatal and obstetrics medical records. Information on BMI at T1 and season of conception (December–May or June–November) was obtained from the medical records. Season was coded as a binary variable, since this explained 30% of vitamin D status in a previous study in pregnant women at the same latitude [16]. Data on education level (≤primary level, secondary level or university level) and origin (continent of birth: Northern Europe, Continental Europe, America, Asia, Africa) were collected from study questionnaires.

### Statistical analysis

Concentrations of 25OHD at T1 and T3 were used as categorical variables, grouped into 25OHD < 30 (used as reference category), 30–49.9, 50–74.9, 75–99.9 and ≥ 100 nmol/L. These groups were chosen to study whether vitamin D insufficiency (30–50 nmol/L), sufficiency (≥ 50 nmol/L) or high status (≥ 75 or ≥ 100 nmol/L) was associated with the outcomes studied, compared to vitamin D deficiency (< 30 nmol/L). Quartiles were not used because the distribution of 25OHD was different at T1 and T3. T1 25OHD was also investigated as a continuous variable as there were few cases in some categories. Change in 25OHD was calculated as the difference between T3 and T1 (T3-T1) and was coded into 3 groups: decrease in 25OHD (≤ 0 nmol/L), small increase (0.1–29.99 nmol/L) or large increase (≥30 nmol/L).

Multivariable logistic regression analyses of the outcome variables SGA, LBW and preterm delivery were performed with 25OHD at T1, T3 and change in 25OHD during pregnancy as the independent variables. For SGA, appropriate for gestational age and large for gestational age were combined as reference. Potential confounders for the associations studied were identified using directed acyclic graphs (www.dagitty.net) [17]. Variables BMI, season of conception, education level and origin were identified and included in the final models. Tobacco use and vitamin D supplement use were also investigated as potential confounders but did not show any confounding effect and were thus not included in the final models. In the multivariable analysis with change in 25OHD as the independent variable, T1 25OHD was also included as a confounder. Correlation between continuous T1 25OHD and continuous change in 25OHD was low (*r* = − 0.22, *p* < 0.001) and it was therefore considered acceptable to include both variables in the same model. Multivariable logistic regression analysis of the outcome pregnancy loss was also performed with 25OHD at T1 (continuous and categorical) as independent variable. These models were adjusted for BMI, season of conception, education level, origin and gestational age at registration to antenatal care (based on last menstrual period). Unadjusted logistic regression analysis was used to assess the association between T1 25OHD (< 30 nmol/L; no/yes) and IUFD. Here, no confounders were included due to the small number of cases (*N* = 9). Significance was accepted at *p* < 0.05. Computer software IBM SPSS Statistics version 22.0 was used for all statistical analyses.

## Results

**Fig. 1:**
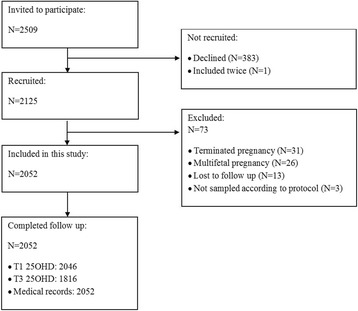
Flow chart of the study inclusion

In total, 2052 women were included in this study, 2046 with a blood sample at T1 and 1816 at T3 (Fig. 1). In total, 1810 women had samples collected both in T1 and T3. Characteristics of the women and the live born infants are shown in Table 1. Mean infant birth weight was 3542 (538) grams and mean gestational age at delivery was 280 (12.4) days. Mean (SD) maternal 25OHD at T1 was 64 (24.4) nmol/L and 75 (34.4) nmol/L at T3. At T1, 10% had 25OHD concentrations < 30 nmol/L and 9% at T3 (Table 2).

**Table 1:** Characteristics of the pregnant women and their infants at birth

	Mean	SD
Birth weight (grams)^a^	3542	537.7
Birth length (cm)^a^	50	2.3
Gestational age at delivery (days)	280	12.4
Gestational age T1 (days)	76	13.8
Gestational age T3 (days)	234	12.9
Maternal s-25OHD T1 (nmol/L)	64	24.4
Maternal s-25OHD T3 (nmol/L)	75	34.4
	N (%)	
Male gender of infant^a^	976 (49.8)	
Small for gestational age (weight or length)	93 (4.5)	
Low birth weight (< 2500 g)	58 (2.8)	
Preterm delivery (< 37 weeks)	78 (3.8)	
Spontaneous preterm delivery (< 37 weeks)	55 (2.7)	
Pregnancy loss	97 (4.7)	
Intrauterine fetal death (≥ 22 weeks)	9 (0.5)	

*T1* first trimester, *T3* third trimester, *25OHD* 25-hydroxyvitamin D

^a^Live born infants only

**Table 2:** The pregnant women’s vitamin D status in the first (T1) and third (T3) trimester of pregnancy, and their characteristics (mean or percent) grouped by 25OHD concentration in (T1)

T1 25OHD (nmol/L)	< 30	30–49.9	50–74.9	75–99.9	≥ 100
N	198	291	788	565	125
%	10.1	14.8	40.1	28.7	6.4
BMI T1 (kg/m^2^)	25.1	24.9	24.4	23.9	24.4
Age T1 (years)	29.4	30.9	31.6	32.0	32.4
Born in Sweden (%)	14.1	54.6	83.3	87.8	93.5
Tobacco use T1 (%)	6.8	4.6	4.0	4.6	1.6
Nulliparous T1 (%)	34.8	42.0	42.6	43.1	42.7
Vitamin D supplement use T1 (%)	10.6	28.2	45.2	56.6	62.9
University level education (%)	32.3	54.9	61.3	67.3	66.1
Small for gestational age (%)	5.7	4.2	5.3	4.1	4.0
Preterm delivery (%)	4.1	5.2	3.8	3.9	1.6
Low birth weight (%)	3.0	4.5	3.1	2.1	1.6
Pregnancy loss (%)	6.3	5.6	4.5	5.1	0
T3 25OHD (nmol/L)	< 30	30–49.9	50–74.9	75–99.9	≥ 100
N	163	330	473	409	443
%	9.0	18.2	26.0	22.5	24.4
Small for gestational age (%)	7.5	5.5	4.7	4.6	2.3
Preterm delivery (%)	4.3	1.2	3.6	2.2	1.6
Low birth weight (%)	4.3	0.3	2.7	1.2	1.4

*T1* first trimester, *T3* third trimester, *25OHD* 25-hydroxyvitamin D

### Birth size and preterm delivery

In total, 93 (4.5%) infants were born SGA. Of the SGA deliveries, 37 were SGA by weight and 56 by length. Also, 58 (2.8%) infants had LBW, while 78 (3.8%) infants were delivered preterm (Table 1). Of those who delivered preterm, 10 delivered before gestational week 31 and thus before the T3 blood sample could be drawn. Among these women, one (10%) had 25OHD concentration < 30 nmol/L in T1.

There were no associations between the predictor T1 25OHD and the outcomes SGA, LBW and preterm delivery (Table 3). Women with T3 25OHD ≥ 100 nmol/L had a lower OR for SGA (OR = 0.32, *p* = 0.031) compared to those with vitamin D deficiency (< 30 nmol/L). Women with T3 25OHD concentrations ≥ 100 nmol/L and 30–50 nmol/L had lower OR for LBW (OR = 0.22, *p* = 0.046 and OR = 0.07, *p* = 0.017, respectively), compared to vitamin D deficiency. However, T3 25OHD was not significantly associated with preterm delivery. The results were not meaningfully affected by additional adjustment for tobacco use or vitamin D supplementation or by excluding cases of preeclampsia (data not shown).

**Table 3:** Association between vitamin D status in pregnancy with birth size and pregnancy loss (adjusted logistic regression analysis)

	Small for gestational age^a^		Preterm delivery (< 37 weeks)^b^		Low birth weight (< 2500 g)^c^		Pregnancy loss^d^	
	OR	CI 95%	OR	CI 95%	OR	CI 95%	OR	CI 95%
T1 25OHD^e^								
Continuous nmol/L	1.003	0.99–1.01	0.996	0.99–1.01	0.999	0.99–1.01	0.989*	0.98–1.00
< 30 (ref)	1.0		1.0		1.0		1.0	
30–49.9	1.078	0.44–2.66	1.647	0.63–4.33	2.549	0.88–7.38	1.030	0.44–2.41
50–74.9	1.632	0.69–3.85	1.260	0.47–3.36	2.287	0.77–6.83	0.688	0.29–1.62
75–99-9	1.292	0.51–3.27	1.332	0.48–3.73	1.665	0.51–5.49	0.809	0.33–1.96
≥ 100	1.294	0.38–4.42	0.502	0.09–2.72	1.255	0.22–7.27	0.000^f^	
T3 25OHD^e^								
< 30 (ref)	1.0		1.0		1.0			
30–49.9	0.771	0.34–1.75	0.302	0.08–1.14	0.071*	0.01–0.63		
50–74.9	0.623	0.27–1.45	0.933	0.31–2.79	0.584	0.17–1.98		
75–99-9	0.657	0.27–1.61	0.614	0.18–2.14	0.237	0.06–1.01		
≥ 100	0.318*	0.11–0.90	0.446	0.11–1.76	0.215*	0.05–0.97		
Δ25OHD^g^								
Large increase (≥ 30 nmol/L) (ref)	1.0		1.0		1.0			
Small increase (0–30 nmol/L)	2.564*	1.16–5.67	2.905*	1.01–8.34	3.122	0.97–10.09		
Decrease (< 0 nmol/L)	3.679**	1.60–8.44	2.894	0.95–8.85	4.722*	1.37–16.28		

*T1* first trimester, *T3* third trimester, *25OHD* 25-hydroxyvitamin D

*p < 0.05 **p < 0.01

^a^Models include 92 (T1) and 80 (T3 and Δ25OHD) cases of small for gestational age

^b^Models include 77 (T1) and 43 (T3 and Δ25OHD) cases of preterm delivery

^c^Models include 57 (T1) and 31 (T3 and Δ25OHD) cases of low birth weight

^d^Models include 96 cases of pregnancy loss

^e^Adjusted for education, origin, season of conception and BMI at T1. Pregnancy loss models are also adjusted for gestational age at registration for antenatal care

^f^No case of pregnancy loss in category

^g^Adjusted for education, origin, season of conception, BMI at T1 and 25OHD at T1

Vitamin D status trajectory between T1 and T3 was inversely related to SGA, preterm and LBW (Table 3). Compared to women with a large increase in 25OHD (≥30 nmol/L), women with a decrease in 25OHD had significantly higher OR of having a child born SGA (OR = 3.7, *p* = 0.002), with LBW (OR = 4.7, *p* = 0.014) as well as a trend toward higher odds of preterm delivery (OR = 2.9, *p* = 0.061). Women with a small increase in 25OHD (0–30 nmol/L) had significantly higher OR of SGA (OR = 2.6, *p* = 0.019) and preterm delivery (OR = 2.9, *p* = 0.047) as well as a trend toward significance for LBW (OR = 3.1, *p* = 0.056), compared to women with a large increase. Neither of these results changed after adjustment for tobacco use and vitamin D supplementation at T3 or after excluding cases of preeclampsia.

### Pregnancy loss

The total rate of pregnancy loss was 4.7% and the rate of late pregnancy loss (gestational week ≥ 14 + 0) was 1.5% (Table 1). There were nine cases of IUFD at gestational week ≥ 22 + 0. Overall, lower T1 25OHD (as a continuous but not categorical variable) was associated with pregnancy loss (Table 3). In unadjusted analysis, T1 25OHD was associated with IUFD and women with 25OHD < 30 nmol/L at T1 had more than fourfold higher odds of IUFD (OR = 4.52, *p* = 0.034).

## Discussion

We found that higher vitamin D status among women in late, but not early, pregnancy was associated with lower probability of SGA and LBW. Vitamin D status trajectory during pregnancy was inversely associated with SGA, LBW and preterm delivery. Women with an increase in 25OHD ≥30 nmol/L from T1 to T3 had the lowest odds of SGA, LBW and preterm delivery. We also found that lower vitamin D status in early pregnancy was related to pregnancy loss.

Previous findings from the GraviD study show lower odds of preeclampsia among women with a large increase (≥ 30 nmol/L) in 25OHD [3]. As SGA, LBW and preterm delivery are related to preeclampsia; these results are consistent with the current findings. However, excluding preeclampsia cases from the analysis did not change the results. Thus, preeclampsia does not seem to mediate the associations between vitamin D status trajectory and neonatal birth size or preterm birth. We have previously shown that the determinants of season-corrected change in 25OHD during pregnancy include origin, sun-exposure and dietary as well as supplementary vitamin D intake [18]. Supplements containing vitamin D were used by 43% in T1 and 39% in T3- mainly multivitamins [19]. As the results between change in 25OHD and neonatal birth size and preterm delivery remained after adjustment for season of conception, origin and vitamin D supplementation, vitamin D status trajectory can be suspected to play a role in healthy pregnancy. Vitamin D has been shown to facilitate the transport of nutrients across the placenta [20, 21], which could contribute to fetal growth [22]. Vitamin D status could also facilitate fetal development by regulating placental inflammation [23]. It is also possible that the associations between fetal growth and maternal vitamin D status are due to residual confounding or reverse causation. Whether vitamin D metabolism is altered in placental dysfunction in unclear and warrants further investigation.

Our results indicates that 25OHD at T3 but not at T1 is associated with SGA and LBW, despite better statistical power at T1 due to more 25OHD samples and subsequently more cases of SGA, LBW and preterm birth. This finding is partly consistent with previous studies where 25OHD concentrations of 25–30 nmol/L were associated with higher probability of SGA [5–7]. The study by Burris et al. [6] sampled women in gestational week 26–28, and found a higher OR for SGA than the two studies that sampled women in T1. This could be interpreted as support of our finding that late rather than early pregnancy vitamin D status is the stronger predictor of fetal growth restriction. Our results also concur with findings by Morley et al. that late but not early pregnancy 25OHD was related to gestational length and neonatal birth size [8]. Our results could also indicate that it takes time for changes in vitamin D metabolism to manifest as changes in circulating 25OHD. We did not see a U-shaped association between early pregnancy vitamin D status and SGA, as previously indicated [9]. We found the lowest odds of SGA among women with the highest T3 25OHD concentration, ≥100 nmol/L. A total of 25% of the women had 25OHD concentrations ≥100 nmol/L at T3. Sampling was evenly distributed across the seasons and time of year cannot explain the high proportion with high 25OHD concentrations. Also, vitamin D supplements were used by almost half of the women but most (88%) used multivitamins containing 5–10 μg of vitamin D3. Supplementation can therefore only partly explain the large proportion with high vitamin D status, and other likely contributors are pregnancy associated endocrine changes and possibly lifestyle factors.

To our knowledge, ours is one of the first studies to find associations between vitamin D status and pregnancy loss. The 25OHD concentration has been shown to have immunological effects in women with a history of recurrent pregnancy loss [12]. Two previous studies have investigated but not found any association between 25OHD and miscarriage [5, 13]. In those studies, 25OHD was used as a categorical variable. One earlier study, from Denmark, found that lower vitamin D status in early pregnancy was associated with pregnancy loss in the first trimester [24]. We found that pregnancy loss was associated with lower 25OHD when expressed on a continuous but not categorical scale. As most women with high vitamin D status were born in Sweden and thus more likely to be familiar with the Swedish health-care system, it is possible that they registered earlier for antenatal care and were more likely to report pregnancy loss. However, the models were adjusted for gestational age at registration for antenatal care. Despite few cases of IUFD in the GraviD cohort, our results suggest that vitamin D deficiency in early pregnancy may be linked to IUFD. These results need confirmation, preferably by adjusted statistical analysis as confounding can be expected, which was not possible in our present study due to few cases.

Strengths of this study are that the GraviD cohort is representative of the general pregnant population in terms of origin, education, parity, BMI and tobacco use [25], which increases the external validity of the findings. Also, gestational age at delivery was estimated by routine ultrasound, which is considered more accurate than dating by last menstrual period [26]. A limitation of this study is that information on pregnancy loss, except IUFD, was self-reported. The information on pregnancy loss is likely correct, albeit without conclusive information on the time of fetal demise. Survival analysis could therefore not be performed, as time to event data were missing. In addition, vitamin D status among women who terminated the pregnancy was not assessed. Data on SGA, LBW and preterm delivery were collected from obstetrics charts based on standardized measures. Since there were few cases of SGA based on birth weight alone, the definition of SGA used in this study was based on birth weight and/or length. Among the SGA cases defined by length only, most were close to also meeting the SGA definition for birth weight. Therefore, the definition used in this study is likely to be relevant. Another limitation is that data on physical activity during pregnancy was only available for a subset of the women and was therefore not used.

While the GraviD study indicates an association between T3 25OHD and consequences of placental insufficiency, change in 25OHD during pregnancy might be a stronger predictor of placental dysfunction, as it is associated with SGA, LBW and preterm delivery. The lowest odds of SGA and LBW are found among women with the largest increments in 25OHD (≥ 30 nmol/L). Interestingly, vitamin D status at T1- around the time the maternal blood flow of the placenta is fully developed- is only related to pregnancy loss.

## Conclusion

In conclusion, lower early pregnancy 25OHD was associated with pregnancy loss. High vitamin D status in late, but not early, pregnancy was associated with lower odds of SGA and LBW. Change in 25OHD during pregnancy was associated with SGA, LBW and preterm delivery, with the lowest odds for women with an increment in 25OHD ≥ 30 nmol/L. Both higher late pregnancy vitamin D status and gestational vitamin D status trajectory can be suspected to play a role in healthy pregnancy.

## Abbreviations

25OHD: 25-hydroxyvitamin D
IUFD: Intrauterine fetal death
LBW: Low birth weight
SGA: Small for gestational age
T1: First trimester of pregnancy
T3: Third trimester of pregnancy
